# Microbial Interactions Support the Role of Ambrosia Beetles as Potential Vectors of Dutch Elm Disease

**DOI:** 10.1007/s00248-025-02624-y

**Published:** 2025-11-15

**Authors:** Juan Carlos Cambronero-Heinrichs, Alessia L. Pepori, Francesco Pecori, Giacomo Santoiemma, Giacomo Cavaletto, Alberto Santini, Davide Rassati

**Affiliations:** 1 Department of Agronomy, Food, Natural Resources, Animals and Environment (DAFNAE), University of Padova, Legnaro (PD), Italy; 2 Centro Nacional de Innovaciones Biotecnológicas (CENIBiot), CeNAT-CONARE, San José, Costa Rica; 3 National Research Council, Institute for Sustainable Plant Protection (CNR-IPSP), Sesto Fiorentino, Firenze, Italy

**Keywords:** *Ophiostoma novo-ulmi*, Vector-pathogen interaction, Invasive species, Microbial symbionts

## Abstract

Dutch elm disease (DED) has caused devastating pandemics in natural elm populations across Europe, North America, and Asia. The primary vectors of DED are native elm-associated bark beetles of the genus *Scolytus*. However, there is evidence suggesting that ambrosia beetles may also carry *Ophiostoma novo-ulmi* and potentially transmit it to elm trees. In this study, we explored microbial interactions and host selection mechanisms that could support the role of ambrosia beetles as vectors of *O. novo-ulmi*. Our study showed that females of *Anisandrus dispar*, *Xyleborinus saxesenii*, *Xylosandrus crassiusculus*, and *Xylosandrus germanus* emerging from both DED-infected and healthy elm logs carried *O. novo-ulmi* DNA. Furthermore, we showed that none of the tested ambrosia beetle fungal symbionts was adversely affected by *O. novo-ulmi*, while *Dryadomyces* spp. and the bacterial symbiont *Erwinia* sp. 1C4 partially restricted or inhibited *O. novo-ulmi* growth without fully suppressing it. Overall, these findings provide additional evidence supporting the potential role of ambrosia beetles as vectors of DED and emphasize the need for further research on this understudied insect-pathogen relationship.

**Supplementary Information:**

The online version contains supplementary material available at 10.1007/s00248-025-02624-y.

## Introduction

Dutch elm disease (DED) has triggered a series of pandemics, devastating natural elm (*Ulmus* spp.) populations across Europe, North America, and Asia [1]. In Europe, DED was initially caused by the pathogen *Ophiostoma ulmi*, responsible for the first epidemic in the early twentieth century [2]. A second, more destructive epidemic began in the mid-twentieth century, driven by the more virulent pathogen *Ophiostoma novo-ulmi* [1, 3], which includes two subspecies, *O. novo-ulmi* ssp. *novo-ulmi* and *O. novo-ulmi* ssp. *americana* [4, 5]. A key role in these pandemics has been played by the new association between these invasive pathogenic fungi and the native elm-associated bark beetles (Curculionidae: Scolytinae) in the genus *Scolytus*. These beetles develop in the tree phloem and have become highly efficient vectors of the disease [6–9]. However, there is evidence suggesting that ambrosia beetles (Curculionidae: Scolytinae and Platypodinae), which develop in the tree xylem, may also carry *O. novo-ulmi* [10] and potentially contribute to its transmission to elm trees [11, 12].

Ambrosia beetles (Curculionidae: Scolytinae and Platypodinae) are wood-boring insects that typically excavate galleries in the xylem of their host trees [13]. Several ambrosia beetle species are markedly polyphagous [14], even though preferences for certain host trees are common [15]. In addition, a number of ambrosia beetle species are attracted to host-emitted volatiles, including sesquiterpenoids and ethanol [16–18]. These volatiles are often produced in response to biotic and abiotic stressors, such as infection by *O. novo-ulmi* and other pathogens [19–21], potentially rendering DED-infected trees attractive to opportunistic ambrosia beetles seeking stressed hosts to colonize. This mechanism may lead ambrosia beetles to encounter *O. novo-ulmi* spores and mycelia during gallery excavation, acquire them on their bodies [22, 23], and potentially carry them to uninfected trees [24]. Except for early studies focused on *Xylosandrus germanus* and conducted in controlled conditions [11, 12], empirical evidence supporting these mechanisms remains limited.

Another relevant biological mechanism to understand the potential role of ambrosia beetles as vectors of DED regards the interactions among *O. novo-ulmi* and the ambrosia beetle microbiome. Ambrosia beetles are associated with diverse fungi and bacteria [25–27], which can play crucial roles for the beetle. Nutritional fungi, for example, serve as the primary or sole food source for both larvae and adults [13]. These fungi are carried inside selective organs called mycetangia [28] and cultivated on gallery walls by founder females [13, 27]. Females lay eggs in newly established galleries only if their symbiotic fungi successfully grow [29, 30], a process that may be compromised by the co-presence of *O. novo-ulmi* within the wood. As observed with the use of entomopathogenic fungi [31–33], *O. novo-ulmi* can potentially outcompete ambrosia beetle symbionts, limiting their growth. A negative effect on these nutritional symbionts could prevent successful nest establishment and offspring production, thereby reducing the number of beetles potentially capable of vectoring Dutch elm disease (DED). It is also evident that offspring may become DED-vectors only if they are contaminated by *O. novo-ulmi* spores or mycelia prior to emerging from the maternal nest. This implies that *O. novo-ulmi* material, i.e. spores and hyphae, must still be present on gallery walls during offspring development. However, the growth and persistence of the pathogen can be challenged by inhibitory effects exerted by the microbial associates of ambrosia beetles, including both fungi and bacteria [34]. All these complex interactions are, however, largely unexplored.

In this study, we investigated some of the mechanisms that can support the potential role of ambrosia beetles as vectors of *O. novo-ulmi*. In particular, we (i) assessed the attractiveness towards ambrosia beetles of DED-infected elm logs vs. healthy elm logs with or without the addition of ethanol, which was used to simulate plant stress responses and trigger beetle attacks; (ii) quantified the DNA of *O. novo-ulmi* on ambrosia beetles emerging from DED-infected and healthy logs; and (iii) explored the interactions among *O. novo-ulmi* and microorganisms associated with ambrosia beetles, including fungal symbionts and bacteria. Specifically, we hypothesized that ambrosia beetles are attracted by DED-infected logs, especially when ethanol is also present in the wood. We also hypothesized that individuals emerging from DED-infected logs carry spores of *O. novo-ulmi* acquired during gallery excavation. Finally, we predicted that fungal symbionts of ambrosia beetles would not be negatively affected by *O. novo-ulmi*, and that *O. novo-ulmi* could grow in co-culture with ambrosia beetle fungal and bacterial associates.

## Materials and Methods

### Attractiveness of DED-Infected Elm Logs

In May 2024, 24 logs (diameter: 5.2 ± 1.4 cm; length: 40.0 cm) were cut from *Ulmus glabra* branches in a mixed forest in Vallombrosa, Firenze, Italy (43°44′11″ N; 11°33′33″ E). Twelve logs were taken from healthy elm trees not showing any internal or external symptoms of DED, nor any feeding signs by *Scolytus* beetles or entry holes by ambrosia beetles. In addition, logs were collected when *Scolytus* beetles were still not active. Thus, any potential infection would have occurred within the preceding year, and symptomatic manifestations would have been evident. The remaining twelve logs were obtained from DED-infected trees, showing severe symptoms of the disease, such as leaf yellowing and wilting. Half of the logs from each group (*n* = 6 per group) were randomly assigned to receive a 5% (v/v) aqueous ethanol solution, whereas the remaining logs were treated with water. Ethanol was chosen because it is a well-established attractant for ambrosia beetles [18], while water has no attractive effect [35]. The combination of DED infection and ethanol treatment was used to simulate a scenario in which DED-infected trees also experience additional stressors that induce ethanol production and release. The 5% ethanol concentration was selected based on previous studies that demonstrated its effectiveness in eliciting attacks by some ambrosia beetles [36, 37]. Treatments were applied by pouring either ethanol solution or water into a 10-cm-deep hole (1.5 cm diameter; ~ 17.3 cm^3^ volume) drilled into one end of each log. Separate drills were used for DED-infected and healthy logs to prevent cross-contamination. Holes were then sealed with plastic caps. On 12 June 2024, the logs were deployed in a broadleaf forest (45°17′14″ N; 11°41′9″ E) on the northern slope of Monte Fasolo (260 m a.s.l.), in the Euganean Hills, Veneto, Northeastern Italy. The forest is dominated by *Ostrya carpinifolia* and *Quercus pubescens* Willd., with *Castanea sativa*, *Fraxinus ornus* L., *Robinia pseudoacacia* L., and other tree species, including elms, present at lower densities. The climate is temperate, with a mean annual temperature of ~ 14 °C and annual precipitation of ~ 800 mm.

Using plastic cable ties attached near the sealed hole, logs were suspended ~ 2 m above the ground. The logs were arranged in a completely randomized block design, with each block (*n* = 6﻿) containing one log for each of the four treatment groups (i.e. water-treated healthy log, ethanol-treated healthy log, water-treated DED-infected log, and ethanol-treated DED-infected log). Within each block, logs were spaced ~ 5 m apart. Blocks were spaced ~ 50 m from each other. Treatment holes were replenished weekly with either ethanol solution or water, according to the assigned treatment. On 16 July 2024, all logs were brought back to the lab, and entry holes were counted. Then, logs were individually enclosed in rearing boxes. For five consecutive weeks, boxes were inspected daily, and emerging beetles were collected, identified to species, and counted.

### Investigating the Presence of *O. novo-ulmi* DNA on Ambrosia Beetle Adult Females that Emerged from the Logs

Each beetle that emerged from logs was placed in a sterile plastic vial and stored at − 20 °C prior to DNA extraction. Individuals were then ground individually using a Mixer Mill 300 (Qiagen). DNA was extracted using the EZNA Insect DNA Minikit (Omega Bio-tek), following the manufacturer’s instructions. The quality of extracted DNA was checked by agarose gel electrophoresis and quantified using a Nanodrop ND-1000 spectrophotometer (NanoDrop Technologies). All DNA samples were analyzed using a duplex TaqMan qPCR assay specific for *Ophiostoma novo-ulmi* [38]. Amplifications were performed using the StepOnePlus Real-Time PCR System (Applied Biosystems, Life Science, Foster City, CA, USA). Primers were synthesized by Eurofins Genomics (Ebersberg, Germany) and TaqMan probes by Applied Biosystems (ThermoFisher Scientific). The amount of fungal DNA was quantified for each individual. Data were analyzed using the SDS 1.9 sequence detection software (Applied Biosystems), with manual adjustment to the baseline and fluorescence threshold settings.

### In Vitro Interaction Assays Between *O. novo-ulmi* and Microbial Symbionts of Ambrosia Beetles

To investigate the interactions of *O. novo-ulmi* with microbial symbionts of ambrosia beetles, three *O. novo-ulmi* isolates (Table S1) were cultured on Sabouraud dextrose agar (SDA), either in co-culture with microbial isolates previously obtained from active galleries of ambrosia beetles (Table S1) or in pure culture. These microbes included (i) fungal strains of the genera *Dryadomyces* and *Raffaelea* isolated from galleries of *X. saxesenii*, *X. crassiusculus*, and *X. germanus* [34] (Table S1) and (ii) the bacterial isolate 1C4 (*Erwinia* sp.), obtained from galleries of *X. crassiusculus* [34] (Table S1). Fungal isolates of *Dryadomyces* and *Raffaelea* were selected because they are obligate nutritional symbionts of ambrosia beetles and represent the primary or sole food source for these insects [39]. The *Erwinia* isolate was tested due to its proposed role as a facultative symbiont, potentially protecting ambrosia beetle fungal gardens from antagonistic fungi [34]. The microbial isolates obtained from ambrosia beetle galleries and used in this study were previously identified through DNA barcoding, targeting the 18S rRNA gene for fungi and the 16S rRNA gene for bacteria [34]. In contrast, the *O. novo-ulmi* isolates corresponded to reference strains.

In fungal–fungal interaction assays, each *O. novo-ulmi* isolate was inoculated onto SDA plates opposite to a nutritional symbiont of ambrosia beetles, with the two inocula placed 2 cm from either side of the plate centre (Figs. 2 and 3). Plates containing only pure cultures of each fungal species (Figs. 2 and 3) were included as controls. Bacterial–fungal interaction assays followed the protocol described by Cambronero-Heinrichs et al. [34]. Briefly, two inocula of individual *O. novo-ulmi* strains were placed on opposite sides of the Petri dish in order to delay direct contact, as in the control plates of the fungal–fungal interaction assays. However, in this case, a 50 µL inoculum of an overnight culture of the bacteria (*Erwinia* sp. 1C4) was streaked in the centre, between the two fungal inocula (Fig. 4). Fungal inocula (6-mm agar plugs) were prepared from 10-day-old cultures incubated at 25 °C in the dark. For both fungus–fungus interactions and bacterial–fungal interactions, Petri dishes were incubated for 10 days at 25 °C in the dark. Following incubation, the area colonized by each fungal strain was quantified using ImageJ software (NIH). In fungal–fungal interaction assays, growth was measured as the percentage of half the Petri dish occupied by each fungus, with a maximum of 50% indicating complete colonization of one half of the plate. In the bacterial–fungal interaction assays, fungal growth was calculated as the percentage of the total Petri dish area colonized by both inocula of the same fungi. In both assays, the growth of each fungal strain in co-culture was then compared to its growth in pure culture. Each treatment was replicated six times.

### Statistical Analysis

Given the non-normality of the data and the limited number of replicates, non-parametric tests were used for all analyses. All tests were performed using R software [40].

To assess the attractiveness towards ambrosia beetles of DED-infected vs. healthy logs with or without the addition of ethanol, a Friedman test followed by a post-hoc Nemenyi test with single-step *p*-value adjustment was applied. The response variable was the number of entry holes, whereas the treatment (four levels: water-treated healthy log, ethanol-treated healthy log, water-treated DED-infected log, ethanol-treated DED-infected log) was the categorical explanatory variable. Block identity was included as the random factor.

To assess the impact of fungal symbionts on *O. novo-ulmi* isolates, a Dunn’s test was used for each *O. novo-ulmi* isolate (i.e. three different tests). The response variable was the percentage of the half-Petri dish area covered by the *O. novo-ulmi* isolate, whereas the identity of the fungus inoculated on the other half of the Petri dish (seven levels: the same *O. novo-ulmi* isolate or one of the six ambrosia beetle symbionts) was the categorical explanatory variable. Plates in which *O. novo-ulmi* was grown in pure culture were used as the control baseline for comparisons in each test.

To assess the effect of *O. novo-ulmi* isolates on the fungal symbionts, a Dunn’s test was used for each symbiont (i.e. six different tests). The response variable was the percentage of the half-Petri dish area covered by the symbiont, whereas the identity of the fungus inoculated on the other half of the Petri dish (four levels: the same fungal symbiont or one of the three *O. novo-ulmi* isolates) was the categorical explanatory variable. Plates in which the fungal symbiont was grown in pure culture were used as the control baseline for comparisons in each test.

Finally, to test the effect of the bacterial isolate 1C4 (*Erwinia* sp.) on *O. novo-ulmi* isolates, Wilcoxon rank-sum tests were used for each *O. novo-ulmi* isolate (i.e. three different tests) to compare the percentage of fungal colonization between Petri dishes with and without the bacterial inoculum.

## Results

### Attractiveness of DED-Infected Elm Logs

**Fig. 1:**
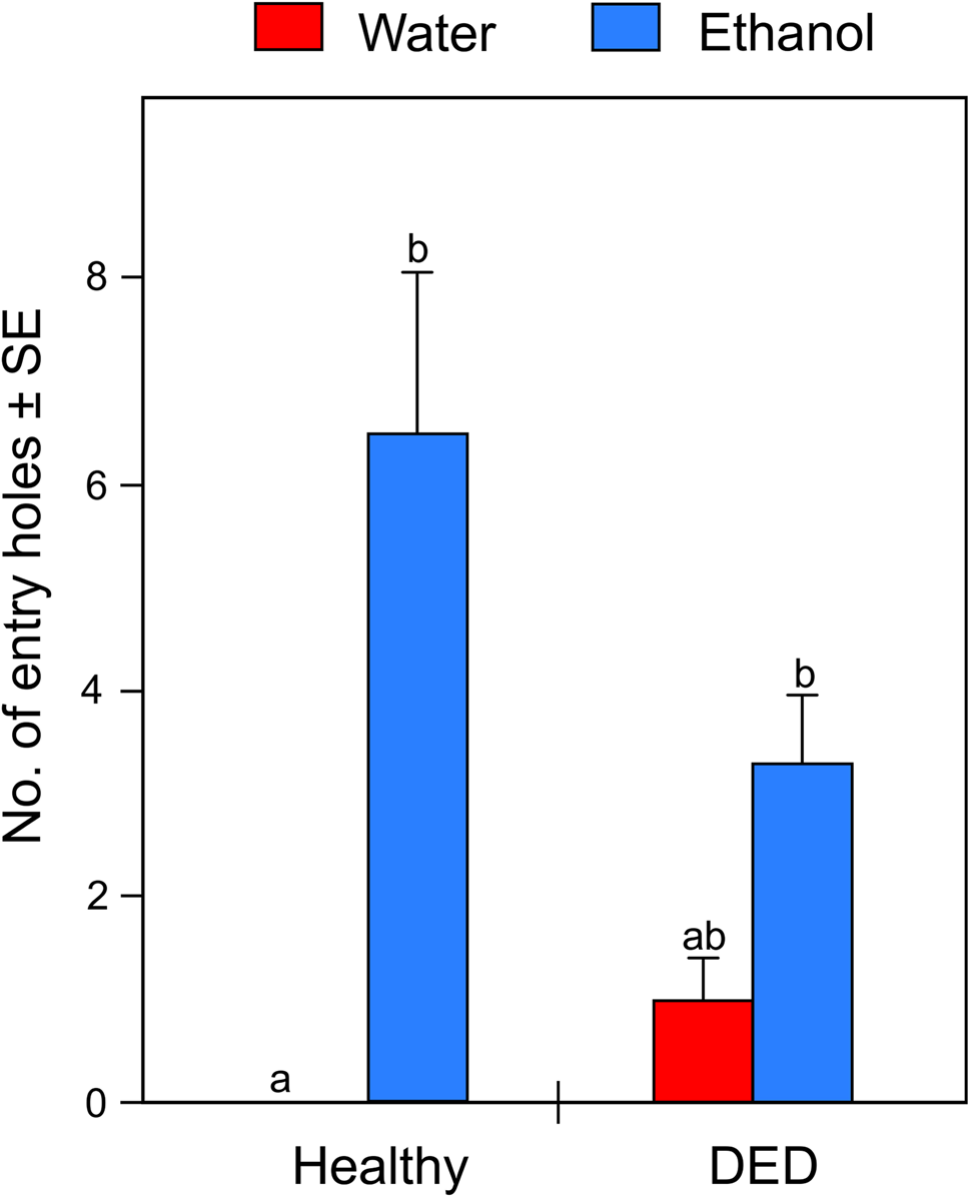
Mean number of entry holes (± SE) observed on healthy and DED-infected logs treated with water or ethanol solution. Different letters above error bars indicate statistically significant differences in pairwise comparisons among treatments (*p*-value < 0.05) based on post-hoc Nemenyi tests (*p*-values reported in Table 1)

A total of 65 entry holes were recorded. The mean number of ambrosia beetle entry holes per log was significantly affected by the treatment (Friedman ${\chi }_{3}^{2}$ = 13.842, *p*-value = 0.003). In particular, the mean number of entry holes was higher in both ethanol-filled healthy-logs (6.50 ± 1.59) and ethanol-filled DED-infected logs (3.33 ± 0.76) than water-treated healthy-logs (0.00 ± 0.00), whereas water-filled DED-infected logs (1.00 ± 0.37) did not differ significantly from the other treatments (Table 1, Fig. 1).

**Table 1:** *P*-values (bolded if < 0.05) from the post-hoc Nemenyi test (single-step *p*-value adjustment) following the Friedman test on the attractiveness of healthy and DED-infected logs treated with water or ethanol solution to ambrosia beetles

	Healthy + ethanol	Healthy + water	DED-infected + water
Healthy + water	**0.004**	-	-
DED-infected + water	0.145	0.608	-
DED-infected + ethanol	0.862	**0.049**	0.536

### Investigating the Presence of *O. novo-ulmi* DNA on Ambrosia Beetle Adult Females that Emerged from the Logs

A total of 25 adult females emerged from ethanol-treated logs, whereas no beetle emerged from water-treated logs (Table 2). Of these, 12 individuals emerged from DED-infected logs and 13 from healthy logs (Table 2). These adult females belonged to four ambrosia beetle species, i.e. the native *Anisandrus dispar* (*n* = 1) and *Xyleborinus saxesenii* (*n* = 3) and the non-native *Xylosandrus crassiusculus* (*n* = 13) and *Xylosandrus germanus* (*n* = 8) (Table 2).

**Table 2:** Mean quantity (± SE) of *Ophiostoma novo-ulmi* DNA (pg/100 mg of tissue) detected in ambrosia beetle individuals that emerged from healthy and DED-infected logs treated with either water or 5% ethanol solution

Ambrosia beetle species	Type of logs	Treatment	No. of individuals	*O. novo-ulmi*
*Anisandrus dispar*	Healthy	Water	-	-
	Healthy	Ethanol	1	65.00
	DED-infected	Water	-	-
	DED-infected	Ethanol	-	-
*Xyleborinus saxesenii*	Healthy	Water	-	-
	Healthy	Ethanol	2	93.00 ± 80.30
	DED-infected	Water	-	-
	DED-infected	Ethanol	1	23.90
*Xylosandrus crassiusculus*	Healthy	Water	-	-
	Healthy	Ethanol	3	766.40 ± 269.40
	DED-infected	Water	-	-
	DED-infected	Ethanol	10	1232.10 ± 197.20
*Xylosandrus germanus*	Healthy	Water	-	-
	Healthy	Ethanol	7	113.70 ± 35.50
	DED-infected	Water	-	-
	DED-infected	Ethanol	1	691.30

DNA of *O. novo-ulmi* was detected on all ambrosia beetle individuals. For both *X. crassiusculus* and *X. germanus*, the amount of DNA present on adult females emerged from DED-infected logs was generally higher than the amount of DNA present on adult females emerged from healthy logs, even though this trend was not valid for *X. saxesenii* (Table 2). Nonetheless, the low number of individuals obtained for some treatments did not allow us to carry out statistical analysis.

### In Vitro Interaction Assays Between *O. novo-ulmi* and Microbial Symbionts of Ambrosia Beetles

**Fig. 2:**
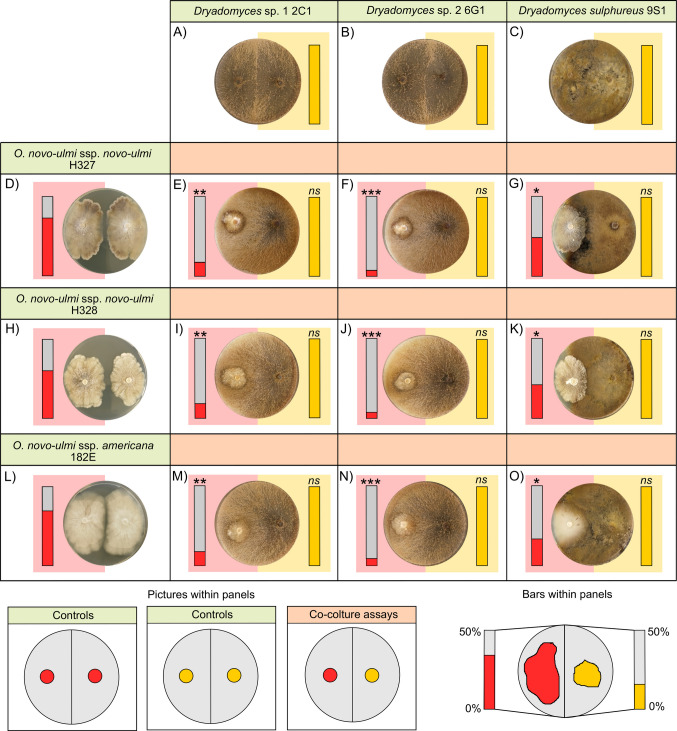
Results of interaction assays between *Ophiostoma novo-ulmi* and fungal symbionts of ambrosia beetles belonging to the genus *Dryadomyces*. Panels with green headings (**A**–**C**, **D**, **H**, **L**) show representative images of controls (i.e. pure cultures), whereas panels with orange headings (**E**–**G**, **I**–**K**, **M**–**O**) display representative images of the interaction assays (co-cultures). Bars within each panel represent the mean percentage of half-Petri dish area covered by the inoculated fungi, ranging from 0 to 50%. Bars on the left side correspond to *O. novo-ulmi* isolates (red), and bars on the right side correspond to *Dryadomyces* spp. (yellow). Asterisks above bars indicate statistically significant differences in fungal growth between co-culture and pure culture conditions. Dunn’s test *p*-values: ∗∗∗  =  < 0.001; ∗∗  = 0.001–0.01; * = 0.01–0.05; *ns* = not significant (> 0.05)

**Fig. 3:**
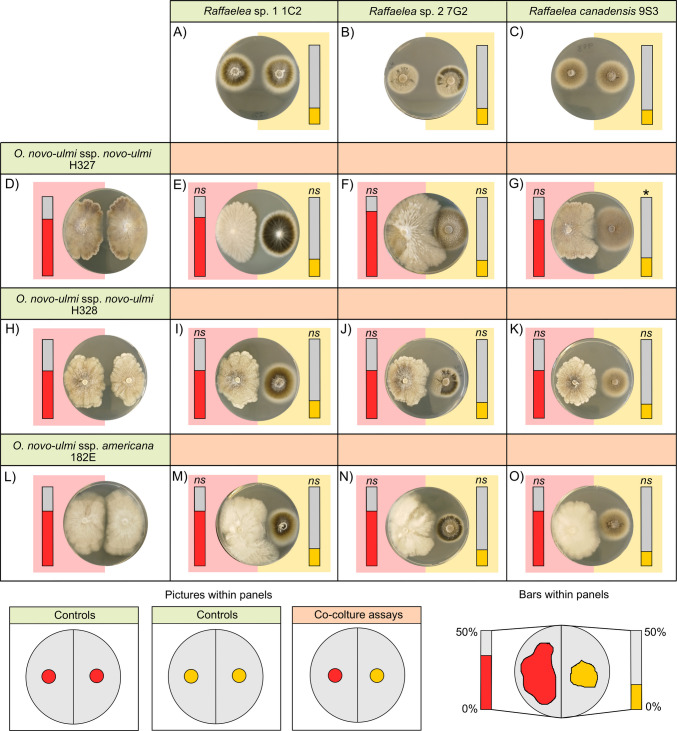
Results of interaction assays between *Ophiostoma novo-ulmi* and fungal symbionts of ambrosia beetles belonging to the genus *Raffaelea*. Panels with green headings (**A**–**C**, **D**, **H**, **L**) show representative images of control plates (pure cultures), while panels with orange headings (E–G, I–K, M–O) display representative images of co-culture assays. Bars within each panel represent the mean percentage of half-Petri dish area covered by the inoculated fungi, ranging from 0 to 50%. Bars on the left correspond to *O. novo-ulmi* isolates (red), and bars on the right correspond to *Raffaelea* spp. (yellow). Asterisks above the bars indicate statistically significant differences in growth between co-culture and control conditions. Dunn’s test *p*-values: * = 0.01–0.05; *ns* = not significant (> 0.05)

None of the six fungal symbionts of ambrosia beetles was negatively affected by the presence of *O. novo-ulmi* isolates (Table 3, Figs. 2 and 3). Specifically, after the incubation period, *Dryadomyces* isolates colonized the entire surface of the half Petri dish where they were inoculated (50.00 ± 0.00%), showing no significant differences compared to their respective pure culture controls (Table 3, Fig. 2). Notably, *Dryadomyces* isolates were also able to partially colonize the opposite half of the Petri dish (Fig. 2). *Raffaelea* isolates co-cultured with the different *O. novo-ulmi* strains did not show significant differences from pure cultures, except for *Raffaelea canadensis* 9S3 in interaction with *O. novo-ulmi* ssp. *novo-ulmi* H327, which exhibited a significantly increased growth compared to its control (Table 3, Fig. 3).

**Table 3:** Mean percentage (± SE) of half-Petri dish area colonized by fungal symbionts of ambrosia beetles when grown in pure culture (control) or in co-culture with one of three *Ophiostoma novo-ulmi* isolates. Results from Dunn’s post hoc tests comparing fungal growth in co-culture versus control are provided. *p*-values: ∗  = 0.01–0.05; *ns* = not significant (> 0.05)

Fungus	Co-cultured fungus	Mean ± SE	*p*-value
*Dryadomyces* sp. 1 2C1	Control	50.00 ± 0.00	**-**
	*O. novo-ulmi* ssp. *novo-ulmi* H327	50.00 ± 0.00	ns
	*O. novo-ulmi* ssp. *novo-ulmi* H328	50.00 ± 0.00	ns
	*O. novo-ulmi* ssp. *americana* 182E	50.00 ± 0.00	ns
*Dryadomyces* sp. 2 6G1	Control	50.00 ± 0.00	**-**
	*O. novo-ulmi* ssp. *novo-ulmi* H327	50.00 ± 0.00	ns
	*O. novo-ulmi* ssp. *novo-ulmi* H328	50.00 ± 0.00	ns
	*O. novo-ulmi* ssp. *americana* 182E	50.00 ± 0.00	ns
*Dryadomyces sulphureus* 9S1	Control	50.00 ± 0.00	**-**
	*O. novo-ulmi* ssp. *novo-ulmi* H327	50.00 ± 0.00	ns
	*O. novo-ulmi* ssp. *novo-ulmi* H328	50.00 ± 0.00	ns
	*O. novo-ulmi* ssp. *americana* 182E	50.00 ± 0.00	ns
*Raffaelea* sp. 1 1C2	Control	15.28 ± 0.56	**-**
	*O. novo-ulmi* ssp. *novo-ulmi* H327	16.28 ± 1.01	ns
	*O. novo-ulmi* ssp. *novo-ulmi* H328	15.76 ± 0.19	ns
	*O. novo-ulmi* ssp. *americana* 182E	15.43 ± 0.32	ns
*Raffaelea* sp. 2 7G2	Control	15.73 ± 0.31	**-**
	*O. novo-ulmi* ssp. *novo-ulmi* H327	15.56 ± 0.31	ns
	*O. novo-ulmi* ssp. *novo-ulmi* H328	15.73 ± 0.24	ns
	*O. novo-ulmi* ssp. *americana* 182E	15.83 ± 0.21	ns
*Raffaelea canadensis* 9S3	Control	14.66 ± 0.71	**-**
	*O. novo-ulmi* ssp. *novo-ulmi* H327	18.21 ± 0.84	*
	*O. novo-ulmi* ssp. *novo-ulmi* H328	15.01 ± 0.62	ns
	*O. novo-ulmi* ssp. *americana* 182E	14.61 ± 0.74	ns

Conversely, *Dryadomyces* isolates negatively affected the growth of all *O. novo-ulmi* strains, as evidenced by a general reduction in the area colonized by the pathogen compared to pure cultures and the partial overgrowth of *Dryadomyces* spp. on *O. novo-ulmi* isolates (Table 4, Fig. 2). *Dryadomyces* sp. 2 6G1 caused the strongest inhibition, followed by *Dryadomyces* sp. 1 2C1, while *Dryadomyces sulphureus* 9S1 was the least inhibitory (Table 4, Fig. 2). In contrast, none of the *Raffaelea* isolates significantly affected the growth of any of the *O. novo-ulmi* isolates (Table 4, Fig. 3).

**Table 4:** Mean percentage (± SE) of half-Petri dish area colonized by the three *Ophiostoma novo-ulmi* isolates when grown in pure culture (control) or in co-culture with fungal symbionts of ambrosia beetles. Results from Dunn’s post hoc tests comparing *O. novo-ulmi* growth in co-culture versus control are reported. *p*-values: ∗∗∗  < 0.001; ∗∗  = 0.001–0.01; ∗  = 0.01–0.05; *ns* = not significant (> 0.05)

Fungus	Co-cultured fungus	Mean ± SE	*p*-value
*O. novo-ulmi* ssp. *novo-ulmi* H327	Control	34.33 ± 0.23	-
	*Dryadomyces* sp. 1 2C1	10.53 ± 0.18	**
	*Dryadomyces* sp. 2 6G1	6.91 ± 0.15	***
	*Dryadomyces sulphureus* 9S1	23.96 ± 0.26	*
	*Raffaelea* sp. 1 1C2	34.03 ± 0.53	ns
	*Raffaelea* sp. 2 7G2	41.30 ± 3.45	ns
	*Raffaelea canadensis* 9S3	35.11 ± 0.65	ns
*O. novo-ulmi* ssp. *novo-ulmi* H328	Control	29.15 ± 0.73	-
	*Dryadomyces* sp. 1 2C1	11.48 ± 0.22	**
	*Dryadomyces* sp. 2 6G1	7.71 ± 0.20	***
	*Dryadomyces sulphureus* 9S1	24.20 ± 0.26	*
	*Raffaelea* sp. 1 1C2	29.05 ± 0.25	ns
	*Raffaelea* sp. 2 7G2	29.58 ± 0.40	ns
	*Raffaelea canadensis* 9S3	29.51 ± 0.25	ns
*O. novo-ulmi* ssp. *americana* 182E	Control	37.95 ± 0.10	-
	*Dryadomyces* sp. 1 2C1	10.35 ± 0.66	**
	*Dryadomyces* sp. 2 6G1	8.15 ± 0.10	***
	*Dryadomyces sulphureus* 9S1	18.45 ± 0.27	*
	*Raffaelea* sp. 1 1C2	38.00 ± 0.27	ns
	*Raffaelea* sp. 2 7G2	38.01 ± 0.20	ns
	*Raffaelea canadensis* 9S3	37.93 ± 0.13	ns

**Fig. 4:**
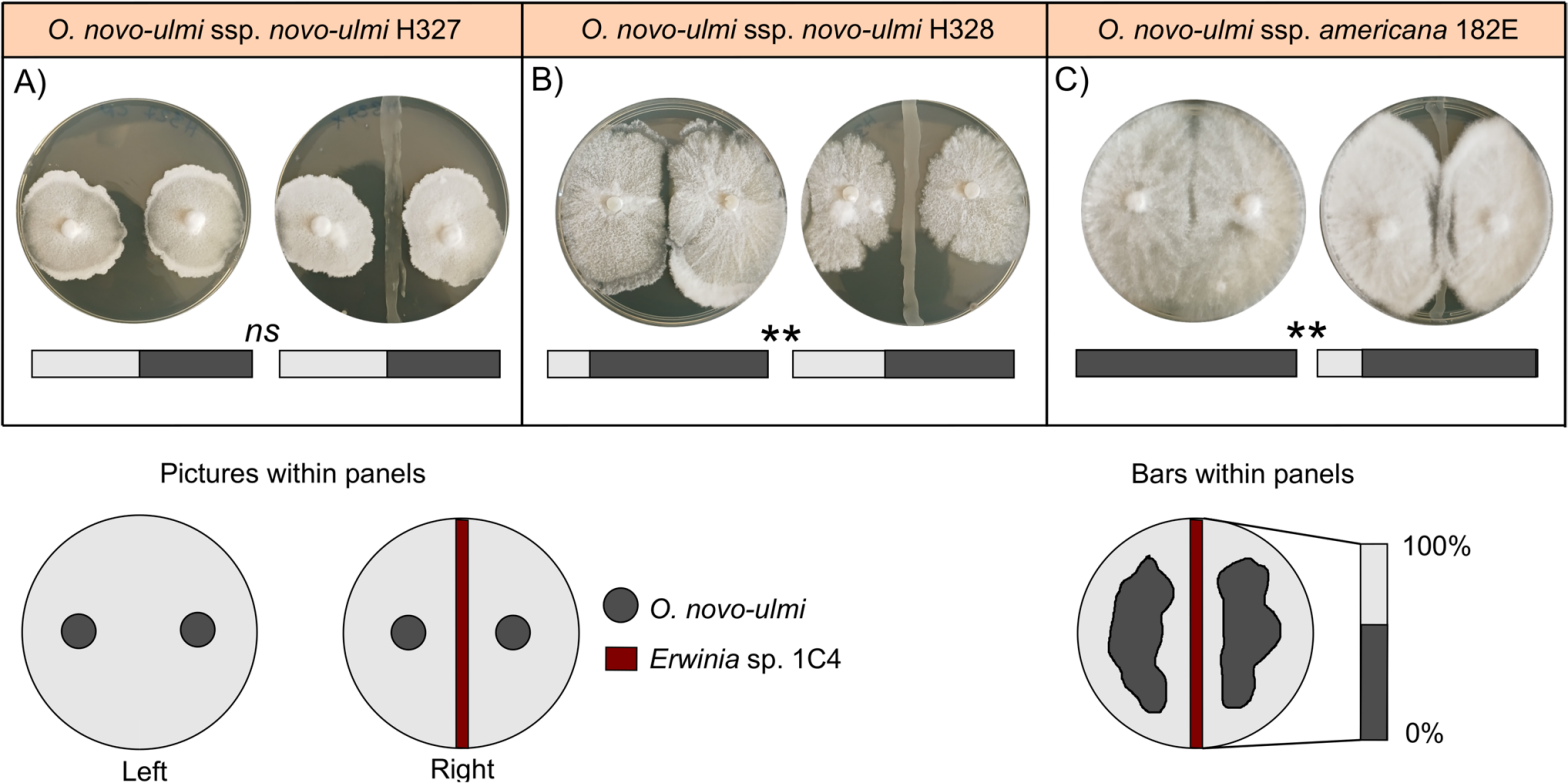
Results of interaction assays between the bacterial isolate *Erwinia* sp. 1C4 and the three *Ophiostoma novo-ulmi* isolates. Each panel (**A**-**C**) shows representative images of the control condition (left) and the co-culture with the bacterium (right), along with bars representing the mean percentage of Petri dish area colonized by the fungus (dark grey section). Asterisks above the bars indicate statistically significant differences in fungal growth between treatments. Wilcoxon rank-sum test *p*-values: ∗∗  = 0.01–0.001; *ns* = not significant (> 0.05)

In the bacterial–fungal interaction assays, the *Erwinia* sp. isolate 1C4 significantly inhibited the growth of *O. novo-ulmi* ssp. *novo-ulmi* H328 and *O. novo-ulmi* ssp. *americana* 182E, as evidenced by the clear reduction in the growth surrounding the bacterial streak (Table 5, Fig. 4). However, no inhibitory effect was observed on *O. novo-ulmi* ssp. *novo-ulmi* H327 (Table 5, Fig. 4).

**Table 5:** Mean percentage (± SE) of Petri dish area colonized by *Ophiostoma novo-ulmi* strains in the presence and absence of the bacterial isolate 1C4 (obtained from active galleries of *Xylosandrus crassiusculus*). Results from the Wilcoxon rank-sum tests are reported. *p*-values: ∗∗  = 0.001–0.01; *ns* = not significant (> 0.05)

Fungal species	Without *Erwinia* sp. 1C4	With *Erwinia* sp. 1C4	*p*-value
*O. novo-ulmi* ssp. *novo-ulmi* H327	49.98 ± 0.60	49.20 ± 0.66	ns
*O. novo-ulmi* ssp. *novo-ulmi* H328	81.88 ± 0.63	57.45 ± 0.34	**
*O. novo-ulmi* ssp. *americana* 182E	100.0 ± 0.0	81.13 ± 0.64	**

## Discussion

Numerous native and non-native ambrosia beetle species are present in Europe, North America, and Asia where DED is still spreading. Their preference for stressed trees [13, 18] makes them strong candidates to vector *O. novo-ulmi*. Although we did not demonstrate a strong attraction of ambrosia beetles to DED-infected logs, our study confirms that these beetles carry *O. novo-ulmi* fungal materials and provides new insights into the interactions among *O. novo-ulmi* and microbial associates of ambrosia beetles that further support their potential role as vectors of DED.

Although we found that the highest number of attacks occurred on ethanol-treated logs regardless of DED presence, some attacks were also observed on DED-infected logs that were not treated with ethanol. While the strong attraction of ambrosia beetles to ethanol is well established [18, 36, 41], evidence for fungal infections in plants inducing attacks is limited and has so far been reported only for other pathogens. For example, trees infected by *Phytophthora ramorum*, the causal agent of sudden oak disease, were more attractive to ambrosia beetles than uninfected trees [21, 42]. Similarly, trees infected by *Harringtonia lauricola*, the causal agent of the laurel wilt disease, were found to be colonized by multiple species of ambrosia beetles [23, 43]. These trends are likely linked to the attractive effect of the host volatiles produced by the host tree in the stress response to the pathogen infection [19–21]. In the case of elm trees infected by *O. novo-ulmi*, some of the sesquiterpenoids [19] likely released by DED-infected logs may have attracted some ambrosia beetle females, which, however, mostly preferred ethanol-treated logs. These results likely reflect both the higher attractive power of ethanol over sesquiterpenoids towards ambrosia beetles [44] and the ability of adult female ambrosia beetles to detect wood tissues already colonized by potentially antagonistic fungi [45], thereby avoiding gallery excavation in them.

We also found that all females emerging from the logs carried *O. novo-ulmi*. In addition to the native *A. dispar* and *X. saxesenii*, previously found in association with *O. novo-ulmi* in Estonia [10], our results suggest that *X. crassiusculus* and *X. germanus* are also carrying *O*. *novo-ulmi* fungal material. Notably, the fungal loads detected on some of the individuals we analyzed were up to 100 times higher than those reported in natural Italian populations of *S. multistriatus*, one of the primary vectors of DED in Europe [38, 46]. Considering the low number of individuals that emerged from the logs, it is likely that most of the beetles we analyzed were females which had initiated gallery excavation but abandoned it before oviposition rather than females belonging to a new generation developed inside the logs. Premature gallery abandonment is, in fact, common when wood tissue does not support the growth of the symbiotic fungi [15, 30]. Elm trees affected by Dutch elm disease are present all over Northern Italy, including our study site, and it might be possible that females came into contact with *O. novo-ulmi* spores before attempting to colonize the experimental logs. This would explain why females emerging from both DED-infected and healthy logs were found carrying fungal material of *O. novo-ulmi.*

It is well known that the fungal symbionts of ambrosia beetles inevitably interact with other fungi already present in the colonized woody substrate [e.g. 47], and these interactions can negatively affect the beetle development [31–33, 48], potentially preventing a given beetle species from becoming a vector of a particular fungus. However, our findings suggest that this ecological constraint may not apply in DED-infected trees, as none of the tested ambrosia beetle fungal symbionts was negatively affected by the presence of *O. novo-ulmi*. Furthermore, our results also suggested that the effect of microbial symbionts of ambrosia beetles on *O. novo-ulmi* does not prevent it from potentially surviving and growing within the beetle galleries. In fact, none of the *Raffaelea* isolates significantly affected the growth of any of the *O. novo-ulmi* isolates, and *Dryadomyces* spp. and the bacterial symbiont *Erwinia* sp. 1C4 restricted or inhibited its growth but without completely suppressing it. This suggests that *O. novo-ulmi* fungal material may remain on gallery walls during offspring development, allowing newly developed females to potentially acquire fungal material of the DED pathogen on their bodies before leaving the nest and carrying it to new hosts. It should be noted that this scenario does not consider the active farming and cleaning behaviours carried out by founder females and their offspring inside the nest [27, 49], which might reduce the persistence of *O. novo-ulmi*.

Finally, it is worth highlighting the potential impact of ambrosia beetles on still healthy *U. glabra* populations, an elm species that is not primarily targeted by *Scolytus* spp. Previous studies, in fact, showed that different species and populations of *Scolytus* spp. preferred *Ulmus minor* Mill. over *U. glabra* when offered the choice of feeding on different elm species [50]. This trend seems to be due to an avoidance mechanism more than to an intrinsic resistance to the pathogens, whereby *U. glabra* is less attractive to *Scolytus* beetles than other elm species [50]. In this context, ambrosia beetles may become a threat for those *U. glabra* populations that remain healthy either because they were not attacked by *Scolytus* spp. or because they are located away from the main distributional range of the latter bark beetles.

## Conclusions

Understanding which insects are capable of carrying or vectoring a fungal pathogen causing significant damage to forest and urban trees is fundamental to developing effective monitoring and control strategies. Our study provides additional evidence supporting the potential role of ambrosia beetles as vectors of DED, highlighting the need for further research into this still overlooked insect-pathogen association. While some evidence already exists under laboratory conditions, future studies should investigate whether ambrosia beetles can transmit *O. novo-ulmi* from infected to healthy elm trees of different species under field conditions. Future studies should investigate the presence of *O. novo-ulmi* fungal material on a broader range of ambrosia beetle species and across a larger geographic scale. In addition, it would be crucial to investigate interactions between ambrosia beetle symbionts and *O. novo-ulmi* on an elm-based medium or in living trees, as well as including more fungi and bacteria. The artificial medium used in our study was highly enriched in sugars and may have significantly altered the chemical expression of the tested microorganisms. Finally, considering the key role played by ethanol not only in host selection but also during colonization, it would be relevant to investigate whether the presence of *O. novo-ulmi* alone in wood tissues is sufficient to trigger the establishment of successful nests and the production of offspring. Considering that the number of ambrosia beetle invasions is increasing worldwide and that the ever increasing international trade and climate change will exacerbate this trend, filling these research gaps becomes fundamental to unravel the future of elm trees and DED.

## Supplementary Information

Below is the link to the electronic supplementary material.

ESM 1 Supplementary Material 1 (DOCX 18.1 KB)

## Data Availability

Data will be made available on request.
