# Supplementary material for: Microbial Interactions Support the Role of Ambrosia Beetles as Potential Vectors of Dutch Elm Disease
Source: Microb Ecol. 2025 Nov 15;88:124. doi: 10.1007/s00248-025-02624-y (PMC12619717; doi:10.1007/s00248-025-02624-y)
Supplement: Supplementary file 1 [file 248_2025_2624_MOESM1_ESM.docx]

**SUPPLEMENTARY MATERIALS**

**Table S1.** List of microbial isolates used in this study, including information on their isolation source and geographical origin.

| **Species** | **Isolate code** | **Isolation source** | **Origin** | **Taxon** |
| --- | --- | --- | --- | --- |
| *Ophiostoma novo-ulmi* ssp. *novo-ulmi* | H327 | *Ulmus* sp. | Slovakia | Fungus |
| *Ophiostoma novo-ulmi* ssp. *novo-ulmi* | H328 | *Ulmus* sp. | Russia | Fungus |
| *Ophiostoma novo-ulmi* ssp. *americana* | 182E | *Ulmus* *minor* | Italy | Fungus |
| *Dryadomyces* sp. 1 | 2C1 | Active gallery of *X. crassiusculus* | Italy | Fungus |
| *Dryadomyces* sp. 2 | 6G1 | Active gallery of *X. germanus* | Italy | Fungus |
| *Dryadomyces sulphureus* | 9S1 | Active gallery of *X. saxesenii* | Italy | Fungus |
| *Raffaelea* sp. 1 | 1C2 | Active gallery of *X. crassiusculus* | Italy | Fungus |
| *Raffaelea* sp. 2 | 7G2 | Active gallery of *X. germanus* | Italy | Fungus |
| *Raffaelea canadensis* | 9S3 | Active gallery of *X. saxesenii* | Italy | Fungus |
| *Erwinia* sp. | 1C4 | Active gallery of *X. crassiusculus* | Italy | Bacterium |
